# An Oxidative Stress-Related Gene Signature in Granulosa Cells Is Associated with Ovarian Aging

**DOI:** 10.1155/2022/1070968

**Published:** 2022-11-03

**Authors:** Nuan Lin, Jiazhe Lin, Torsten Plosch, Pingnan Sun, Xiaoling Zhou

**Affiliations:** ^1^Center for Reproductive Medicine, Shantou University Medical College, Shantou 515041, China; ^2^Department of Obstetrics and Gynecology, University of Groningen, University Medical Centre Groningen, 9700 RB Groningen, Netherlands; ^3^Department of Obstetrics and Gynecology, The First Affiliated Hospital of Shantou University Medical College, Shantou 515041, China; ^4^Department of Neurosurgery, The First Affiliated Hospital of Shantou University Medical College, Shantou 515041, China; ^5^Stem Cell Research Center, Shantou University Medical College, Shantou 515041, China; ^6^Guangdong Provincial Key Laboratory of Infectious Diseases and Molecular Immunopathology, Shantou University Medical College, Shantou 515041, China

## Abstract

Ovarian aging is associated with a decrease in fecundity. Increased oxidative stress of granulosa cells (GCs) is an important contributor. We thus asked whether there is an oxidative stress-related gene signature in GCs associated with ovarian aging. Public nonhuman primate (NHP) single-cell transcriptome was processed to identify GC cluster. Then, a GC signature for ovarian aging was established based on six oxidative stress-related differentially expressed genes (*MAPK1*, *STK24*, *AREG*, *ATG7*, *ANXA1*, and *PON2*). Receiver operating characteristic (ROC) analysis confirmed good discriminating capacity in both NHP single-cell and human bulk transcriptome datasets. Gene expression levels were investigated using qPCR in the human ovarian granulosa-like tumor cell line (KGN) and mouse GCs. In an oxidative stress model, KGN cells were treated with menadione (7.5 *μ*M, 24 h) to induce oxidative stress, after which upregulation of *MAPK1*, *STK24*, *ATG7*, *ANXA1*, and *PON2* and downregulation of *AREG* were observed (*p* < 0.05). In an aging model, KGN cells were continuously cultured for 3 months, leading to increased expressions of all genes (*p* < 0.05). In GCs of reproductively aged (8-month-old) Kunming mice, upregulated expression of *Mapk1*, *Stk24*, *Atg7*, and *Pon2* and downregulated expression of *Anxa1* and *Areg* were observed (*p* < 0.01). We therefore here identify a six-gene GC signature associated with oxidative stress and ovarian aging.

## 1. Introduction

The ovary, which serves as the source of oocytes and sex hormones, is indispensable for maintaining female fertility and endocrine homeostasis [1]. However, there is a limited duration of female fertility due to the quantitative and qualitative decline of oocytes with age [2], a physiological phenomenon often referred to as ovarian aging. Ovarian aging is associated with a decrease in fecundity, poor response during hyperstimulation in assisted reproductive technology (ART), increased risk of miscarriage, and chromosomal aneuploidies in the offspring [3]. Ovarian aging generally becomes apparent as early as 35 years of age in women who are destined to undergo menopause, the final stage of the ovarian aging process [4]. However, the highly variable onset of menopause among individual women indicates the great variability of decline in oocyte number and quality [3]. Furthermore, chronological age does not always correspond to biological age, which is crucial in the prediction of response to ovarian stimulation in ART clinical practice [5] and, presumably, oocyte quality associated with conception rates [6]. Thus, individual assessment of ovarian aging at an early stage of reproductive age may be crucial for counseling patients about their chances for pregnancy.

In the vast majority of mammalians, the primordial follicular pool responsible for the female reproductive lifespan and fertility is established during embryonic life and becomes exhausted with aging. Granulosa cells (GCs) play an essential role in the maintenance of the follicle pool [7]. On the one hand, during the embryonic stage, breakdown of the germ cell nest and primordial follicle formation involves the invasion of pre-GCs [8]. On the other hand, follicle depletion during reproductive senescence originates from oocyte demise as a consequence of surrounding GC apoptosis [3]. Aging GCs can cause oocyte dysfunction by interrupting the progress of meiosis during oocyte maturation and acquisition of the full developmental potential of oocytes [9]. McReynolds et al. reported the effects of aging on ovarian cumulus GCs, where women with advanced age had a significantly different cumulus GC proteome compared with younger women [10], indicating that changes in cumulus GC gene expression may reflect ovarian aging. Furthermore, Bosco et al. found that apoptosis and survival molecules in human cumulus GCs can serve as an oocyte competence marker [11, 12].

Reactive oxygen species (ROS) are free radicals that arise from cellular metabolism [13]. Increased endogenous ROS can be generated by mitochondria, highly dynamic organelles that respond to cellular energy demand changes [3]. During follicle growth, especially secondary and tertiary follicle development, increased energy and nutritional demands associated with accelerated metabolic rates occur in rapidly proliferating GCs, resulting in increased ROS production as well as oxidative stress [14]. Alcohol, smoking, radiation, and pathology, such as obesity, also generate a large amount of ROS in GCs [15]. It has recently been reported that GC senescence in mice can be induced by enhanced ROS levels, whereas treatment with melatonin, a potent antioxidant, delays GC and ovarian aging [16]. This was reinforced further by an in vivo mouse study showing that melatonin attenuates ovarian mitochondrial oxidative stress and reduces age-induced fertility decline, with increased follicle numbers, litter size, and telomere length of ovarian cells [17]. Although there is emerging research revealing the critical involvement of GC ROS in ovarian aging [18, 19], few efforts have been invested into searching for a discriminating gene signature for ovarian aging, specifically that which is contributed by GC oxidative stress. In this study, we investigate whether there is an oxidative stress-related gene signature in GCs that is linked to ovarian aging, thereby adding insight into the contribution and potential biomarkers of GC-specific oxidative stress in ovarian aging.

## 2. Materials and Methods

### 2.1. Public Nonhuman Primate (NHP) Single-Cell RNA-seq Data Processing

Single-cell RNA sequencing (scRNA-seq) expression profiles were downloaded from the Gene Expression Omnibus (GEO) database. Data extracted from GSE130664 provided the ovarian tissue RNA expression from 4 young and 4 aged NHPs. For quality control, scRNA-seq data exploration, and statistical analysis, the Seurat package in R 3.5.1 was applied [20]. First, low-quality cells were excluded based on the following quality control standards: (1) genes detected in less than 3 cells, (2) cells with less than 200 total detected genes, and (3) cells with equal to or more than 5% of genes expressed in mitochondria. Next, by applying a linear regression model, gene expression of the remaining cells was normalized. To perform cluster classification analysis across all cells, principal component analysis (PCA) and the *t*-distributed stochastic neighbor embedding (*t-*SNE) algorithm was sequentially applied to identify significantly available dimensions with a *p* < 0.05 and dimensionality reduction with 20 initial principal components, respectively. Within each cell cluster, differential expression analysis among all genes was performed using the limma package in R to determine potential marker genes, with adjusted *p* < 0.05 and |log2(fold change(FC))| > 0.01 set as criteria. Finally, different cell clusters were identified and annotated with known classical markers *AMH*, *WT1*, *INHA*, and *CYP19A1* [19, 21, 22] for GC.

### 2.2. Identification of Oxidative Stress-Related Differentially Expressed Genes (DEGs) between Young and Aged NHPs

To identify DEGs of GCs between young and aged NHPs, the edgeR package was used (R version 3.6.2). Genes with |log2(fold − change)| ≥ 1 and false discovery rate (FDR) < 0.05 were considered as DEGs. Oxidative stress-related genes were extracted from the GO_ OXIDATIVE_STRESS gene set in the Molecular Signatures Database v7.1 website. The DEGs were overlapped with the oxidative stress-related gene set to generate the oxidative stress-related DEGs, which were then used to construct a model to distinguish the aged NHPs from the young using the “glmnet” package, version 3.0-2 in R language. To prevent overfitting of the model, least absolute shrinkage and selection operator (LASSO) regression was performed to further identify the oxidative stress-related DEGs with an independent discriminating value. Next, the area under the curve (AUC) of the receiver operating characteristic curve (ROC), which was implemented by R package “pROC,” version 1.16.2, was used to measure the performance of the classifier. Finally, the dataset (GSE81579) providing RNA-seq of human cumulus GCs from seven aged (>40 years old) and eight young patients (<35 years old) was applied as validation.

### 2.3. Functional Enrichment Analysis and Nomogram Establishment

Gene set enrichment analysis (GSEA) was performed to identify the potential biological functions and pathways in the gene signature, followed by Gene Ontology (GO) and Kyoto Encyclopedia of Genes and Genomes (KEGG) pathways explored. According to the GSEA website, an FDR of 0.25 is reasonable in the setting of exploratory discovery for candidate hypothesis, while a more stringent FDR may lead to overlooking potentially significant results. Thus, gene sets with FDR < 0.25 in the high- and low-score groups in the scRNA-seq dataset were considered significantly different and were selected. A nomogram was constructed based on the six oxidative stress-related genes of GC for discriminating the reproductively aged and the reproductively young.

### 2.4. KGN Cell Line Culture

The KGN cell line, which was established from a human ovarian granulosa-like tumor and expresses typical GC markers, was purchased from Cellcook Biotech Co., Ltd. (Guangzhou, China) and cultured as previously described [23]. Cells were maintained in DMEM/F12 medium (Hyclone/Invitrogen, Carlsbad, CA, USA) supplemented with 10% fetal bovine serum (FBS; Sijiqing, China) and antibiotics (100 IU/ml of penicillin and 100 mg/ml of streptomycin; Gibco BRL/Invitrogen) at 37°C in a humidified environment with 5% CO_2_.

### 2.5. Mouse Granulosa Cell (mGC) Extraction and Culture

All mouse procedures were performed in accordance with the guidelines of the Animal Research Institute Committee at Shantou University Medical College (SUMC2020-384). 8-week-old and 8-month-old female Kunming mice were purchased from the Beijing Vital River Laboratory Animal Technology Co., Ltd. (Beijing, China), group-housed in a temperature-controlled (22 ± 2°C) room with a 12/12 h light/dark cycle (lights on from 7:00 a.m. to 7:00 p.m.), and had ad libitum access to water and food. Female Kunming mice were injected intraperitoneally with 10 units of pregnant mare serum gonadotropin (PMSG), which was purchased from the Ningbo Second Hormone Factory (Ningbo, Zhejiang, China), and sacrificed 36-48 h later. Superovulated mouse ovaries were obtained and transferred to Petri dishes (35 × 15 mm) filled with PBS and then, under a surgical dissecting microscope (Olympus, SZ51, Tokyo, Japan), punctured with a syringe to release the mGCs from the dominant follicles. The cells were cultured in DMEM/F-12 (1 : 1) medium (Hyclone/Invitrogen, Carlsbad, CA, USA) supplemented with 10% FBS (Sijiqing, China) and antibiotics (100 IU/ml of penicillin and 100 mg/ml of streptomycin; Gibco BRL/Invitrogen) at 37°C in a humidified environment with 5% CO_2_ for 4 days.

### 2.6. Oxidative Stress Induction

Menadione is inexpensive and stable and displays low toxicity [24] and was thus applied in our study as an agent to induce oxidative stress in KGN cells. KGN cells were treated with 7.5 *μ*M menadione (A502486-100 g, Sangon Biotech) for 24 hours to induce oxidative stress. N-acetyl-L-cysteine (NAC; 10 mM) (ST1546, Beyotime), which is a powerful antioxidant that acts by directly scavenging free radicals [25], was applied 3 min before and during menadione application.

### 2.7. Detection of Intracellular ROS

Accumulation of intracellular ROS was determined by flow cytometry (Accuri^™^ C6, BD Biosciences, San Jose, CA) using a Reactive Oxygen Species Assay Kit (S0033S, Beyotime). KGN cells were incubated with 10 *μ*M 2′,7′- dichlorodihydrofluorescein diacetate (DCFH-DA) for 20 min at 37°C in the dark and then washed three times with DMEM. KGN cells were then harvested, and DCF fluorescence intensity was detected by flow cytometry at an excitation wavelength of 488 nm and emission wavelength of 525 nm.

### 2.8. Senescence-Associated Beta-Galactosidase (SA-*β*-Gal Assay)

SA-*β*-gal activity is a widely used biomarker of cellular senescence [26] and was thus applied to identify KGN cell and mouse GC senescence. KGN cells and mGCs were seeded on 12-well plates at a density of 5^∗^10^5^ cells/ml, followed by being washed with PBS and fixed and stained with X-gal solution (C0602, Beyotime Biotechnology) overnight at 37°C. Cells were imaged and photographed using an inverted microscope (Axio Observer A1, Zeiss, Germany).

### 2.9. RNA Isolation and Quantitative Real-Time PCR (qRT-PCR)

Total RNA was extracted from KGN cells and mGCs using TRIzol reagent (Invitrogen, Carlsbad, CA, USA). Total RNA (1 *μ*g) was reverse-transcribed into cDNA with an RT-PCR Kit (FSQ-101; Toyobo), and qPCR was performed in triplicate for each sample using 2× Power SYBR Green Master Mix (Applied Biosystems) in an ABI 7500 PCR system (Thermo Fisher Scientific Inc.). The specific primers used for qRT-PCR are listed in Table S1. The concentration of all primers used was 250 nM. The qPCR program consisted of 5 min at 95°C, followed by 40 cycles of 95°C for 10 s and 60°C for 30 s. The fold change of each gene expression was calculated using the 2*ΔΔ*CT method with glyceraldehyde-3-phosphate dehydrogenase (*GAPDH* or *Gapdh*) as the internal control.

### 2.10. Statistical Analysis

All experiments were repeated at least three times, and quantitative data are presented as the mean ± standard deviation. After checking for normal data distribution and homogeneity of variances, using the Shapiro–Wilk and Levene tests, respectively, statistical differences between two groups were examined by the Student *t*-test, and among three groups using one-way ANOVA. Except for the bioinformatic analysis, which was conducted using R programming language, all statistical tests were analyzed by the SPSS version 16.0 software (SPSS, IL, USA). A *p* < 0.05 was considered to indicate statistical significance.

## 3. Results

**Figure 1:**
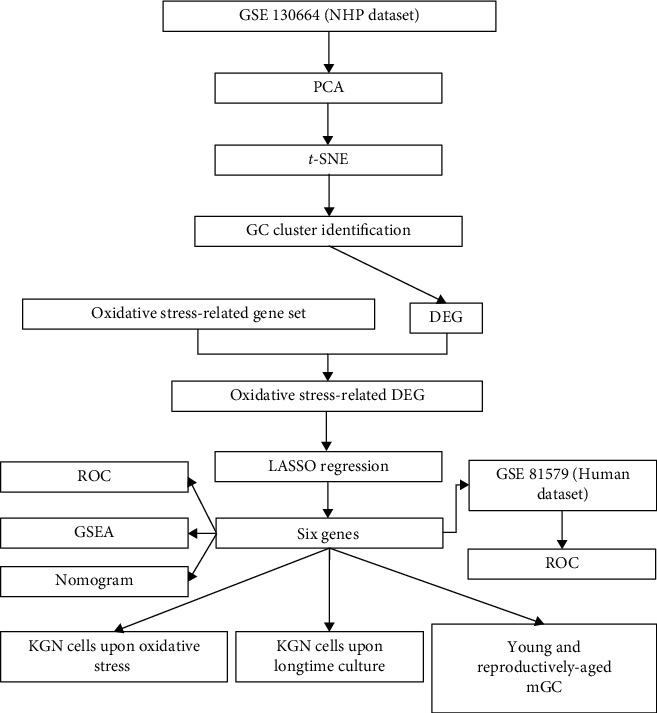
Flowchart shows the steps of this study. Abbreviations: NHP: non-human primate; PCA: principal component analysis; *t*-SNE: *t*-distributed stochastic neighbor embedding; GC: granulosa cell; DEG: differentially expressed gene; LASSO: least absolute shrinkage and selection operator; ROC: receiver operating characteristic curve; GSEA: gene set enrichment analysis; mGC: mouse granulosa cell.

A flowchart of the study design is shown in Figure 1.

### 3.1. Identification of GC Cluster in NHP Ovarian Tissue Using scRNA-seq Data

**Figure 2:**
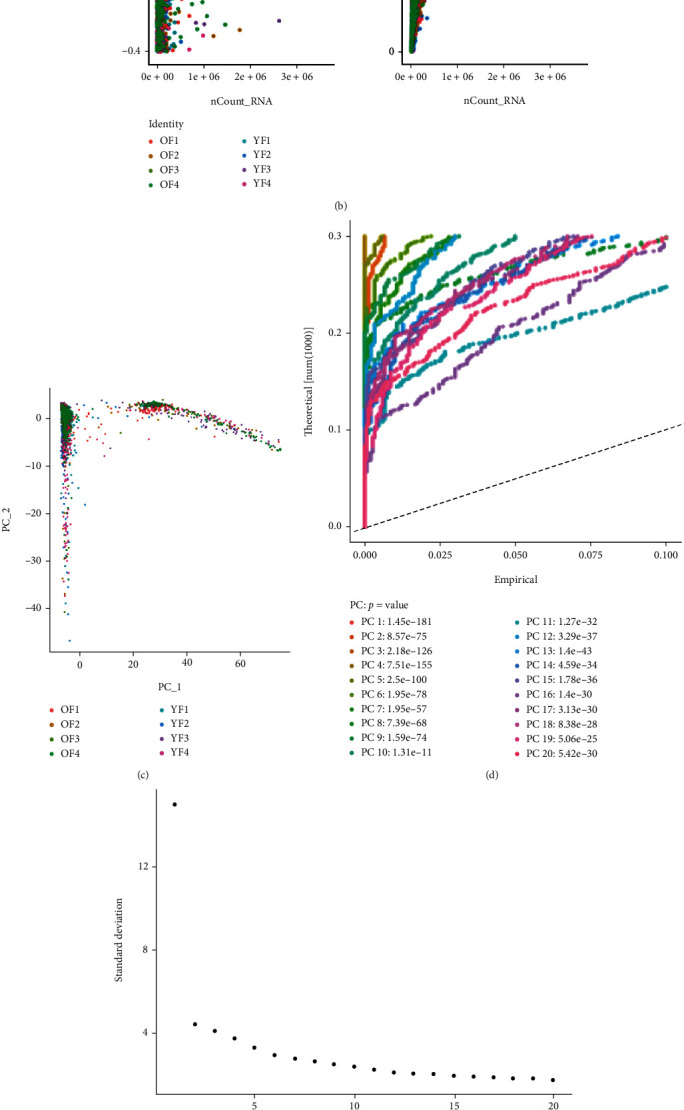
Single-cell RNA-seq analysis of NHP ovarian tissues identifies GC cluster. (a) The number of genes, gene sequences, and the percentage of mitochondrial genes detected in each sample. (b) The relationship between the sequencing depth and the percentage of mitochondrial genes as well as the number of genes. (c) The distribution of cells in each sample after PCA linear dimensionality reduction. (d) *p* value distribution of 20 principal components after PCA linear dimension reduction. (e) Elbow plot shows each principal component. (f) *t*-SNE plot showing 14 ovarian cell types, with cluster 3 corresponding to GCs based on four of the known specific GC markers: *AMH*, *WT1*, *INHA*, and *CYP19A1*.

Low-quality cells were excluded following the quality control standard and the normalization of ovarian cell scRNA-seq data (Figure 2(a)). The number of detected genes and the sequencing depth were significantly related (Figure 2(b)). PCA results did not show clear separations of ovarian cells between young and old NHPs (Figure 2(c)). Twenty principal components were selected, based on an estimated *p* < 0.05, for subsequent analysis (Figure 2(d)). Afterward, the *t*-SNE algorithm was applied, leading to classification of NHP ovarian tissue cells into 14 distinct clusters (clusters 0-13) (Figure 2(e)). Differential gene expression analysis among clusters was performed. Based on four of the known cell-type-specific markers for GCs listed as *AMH*, *WT1*, *INHA*, and *CYP19A1* [19, 21, 22], cluster 3 that corresponded to GCs was annotated (Figure 2(f)).

### 3.2. Construction and Performance Evaluation of an Oxidative Stress-Related GC Gene Signature for Identifying Ovarian Aging

**Figure 3:**
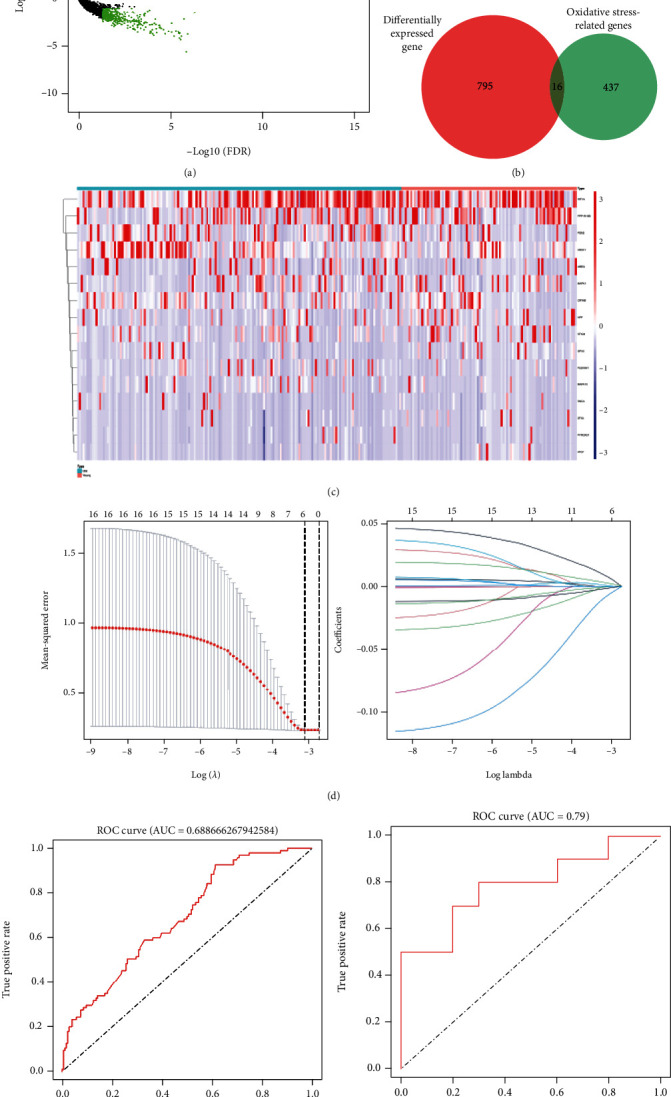
An oxidative stress-related gene signature is built based on 6 DEGs between young and old NHPs and is of good discriminating performance. (a) Volcano map shows the expression of 811 DEGs in NHP GCs. (b) Venn diagram shows overlap of the DEGs and oxidative stress-related genes. (c) Heat map shows 16 oxidative stress-related DEGs in GCs. (d) LASSO regression identifies 6 oxidative stress-related DEGs (*MAPK1*, *STK24*, *AREG*, *ATG7*, *ANXA1*, and *PON2*) with independent discriminating value for ovarian aging. (e) ROC curve of the oxidative-stress related gene signature for ovarian aging in the NHP dataset (AUC = 0.69). (f) ROC curve of the oxidative-stress related gene signature for ovarian aging in a human dataset (AUC = 0.79).

To identify DEGs between old and young NHP GCs, mRNAs from GSE130664 were filtered by edgeR packages. As a result, 811 DEGs from the dataset with |log2(fold − change)| ≥ 1 and FDR < 0.05 are shown by the volcano plot (Figure 3(a)). Following overlap of the 811 DEGs and the gene set containing 453 oxidative stress-related genes, 16 oxidative stress-related DEGs were identified (Figure 3(b)) and their expressions are shown in a heat map (Figure 3(c)). Finally, 6 oxidative stress-related DEGs with independent discriminating capacity in GCs were identified for ovarian aging by LASSO regression (Figure 3(d)). The ovarian aging predicting score formula was as follows:

(1) $$\begin{array}{c} \text{Predicted score}=0.00184\times \text{expression level of }ATG7+\left(-0.0013\right)\times \text{expression level of }ANXA1+\left(-0.000319\right)\times \text{expression level of }PON2+\left(-0.0000677\right)\times \text{expression level of }AREG+0.000181\times \text{expression level of }MAPK1+0.000643\times \text{expression level of }STK24. \end{array}$$

The ROC curve showed that the discriminatory ability of the signature is reasonable (AUC = 0.69) (Figure 3(e)). To construct the signature, the expressions of the six mRNAs were extracted from the human GC dataset (GSE81579), which came from seven aged (>40 years old) and eight young patients (<35 years old). The ROC curve again showed good discriminatory value (AUC = 0.79) (Figure 3(f)).

### 3.3. Functional Analysis and Application of the Six Oxidative Stress-Related Genes

**Figure 4:**
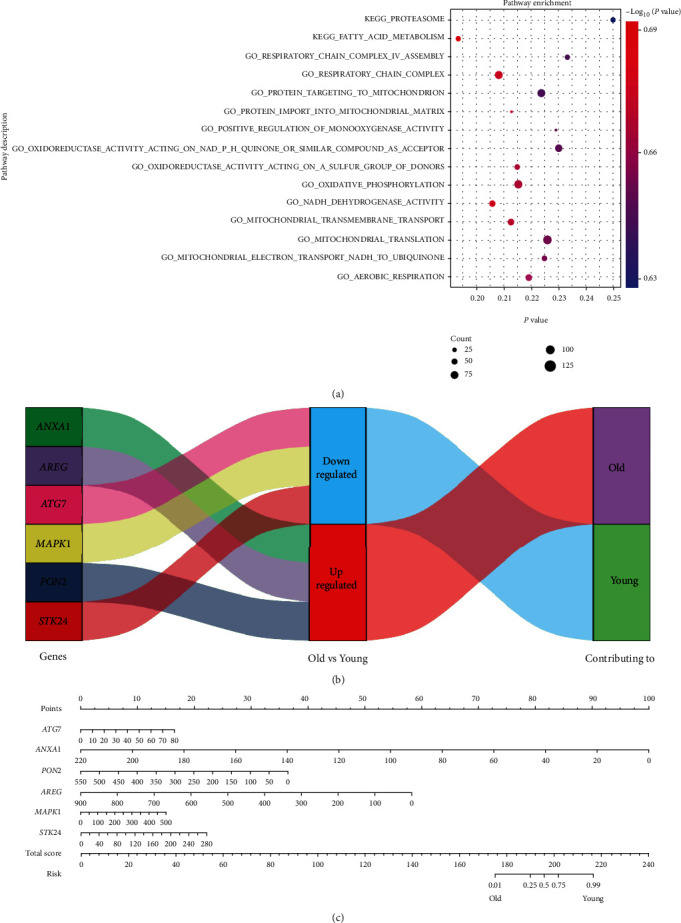
Functional analysis and application of the six oxidative stress-related genes. (a) GSEA shows the gene signature for ovarian aging is associated with oxidative stress-related pathways. (b) A diagram is drawn for showing the correlation between gene expression and their contribution to ovarian aging. The left column shows the six oxidative stress-related genes. The middle column shows the down- or upregulated gene expressions of old NHPs compared to those of the young. The third column shows that the downregulated genes are contributing to a younger phenotype, while the upregulated genes are contributing to an older phenotype. (c) For easier clinical use, a nomogram based on the GC signature is built to visualize the predicted occurrence of ovarian aging.

Following GSEA, 66 GO and KEGG pathways associated with the signature were enriched (FDR < 0.25) (Table S2), including oxidative phosphorylation, mitochondrial transmembrane transport, and positive regulation of monooxygenase (Figure 4(a)). A diagram was drawn to show the correlation between the expression of a gene and its contribution (Figure 4(b)). The left column shows the six genes in NHP GCs. The middle column showed the down- and upregulated gene expressions of GCs in old NHPs compared to the young control. The right column shows that the downregulated genes are contributing to a younger phenotype, whereas the upregulated genes are contributing to an older phenotype. For detailed, fold change of gene expression and the respective factor to the phenotype of being old or young are shown in Table 1. To facilitate clinical application, a nomogram was established (Figure 4(c)).

### 3.4. Expressions of the Six Oxidative Stress-Related Genes in the KGN Cell Line upon Menadione-Induced Oxidative Stress

**Figure 5:**
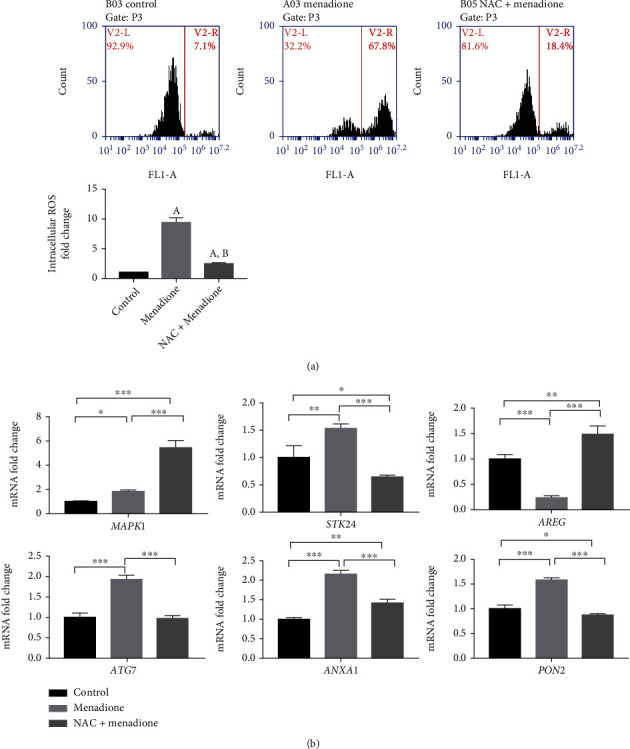
Identification of the menadione-induced oxidative stress KGN cell model and the expression of six oxidative stress-related genes among the control group, menadione group, and NAC+menadione group. (a) Intracellular ROS level of KGN cells among the control group, menadione group, and NAC+menadione group is detected using flow cytometry. (A) indicates *p* < 0.05 compared to the control group, and (B) indicates *p* < 0.05 compared to the menadione group. (b) The expression of six genes in KGN cells among the control group, menadione group, and NAC+menadione group is detected using qPCR, with *GAPDH* as a housekeeping gene. The antioxidant N-acetyl-L-cysteine (NAC; 10 mM) is applied 3 min before and during menadione application. One-way ANOVA is used to detect the significance. Data represent mean ± SD.; *n* = 3 in each group. ^∗^*p* < 0.05, ^∗∗^*p* < 0.01, and ^∗∗∗^*p* < 0.001.

To confirm that menadione induce increases ROS levels in KGN cells, DCF fluorescence intensity was detected by flow cytometry. As shown in Figure 5(a), an increased level of intracellular ROS was observed in the menadione group compared to the control (*p* < 0.05), whereas in the presence of NAC, the ROS level decreased significantly compared with the menadione group (*p* < 0.05). Then, the mRNA expression of the six oxidative stress- related genes in KGN cells in the control group, menadione group, and NAC+menadione group was determined. Upregulated expression of *MAPK1*, *STK24*, *ATG7*, *ANXA1*, and *PON2* and downregulation of *AREG* were observed in the menadione group (*p* < 0.05). Oxidative stress-induced changes were partially (*ANXA1*) or completely (*STK24*, *AREG*, *ATG7*, and *PON2*) abolished in the NAC pretreatment group (*p* < 0.05), while *MAPK1* activation was further increased when cells were also exposed to NAC (*p* < 0.001) (Figure 5(b)).

### 3.5. Expressions of the Six Oxidative Stress-Related Genes Are Aberrantly Different in Replication-Induced Aged Cells Compared to Young Controls

**Figure 6:**
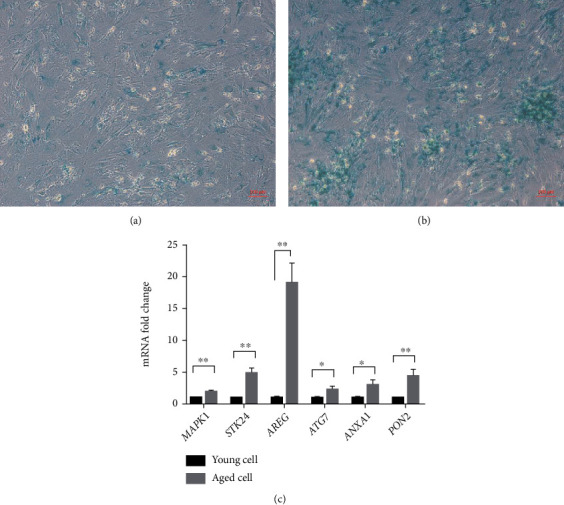
Identification of the replication-induced KGN cell aging model and the expression of six oxidative stress-related genes between young and aged cells. (a) SA-*β*-gal activity in young KGN cells within P5 that was freshly recovered. (b) SA-*β*-gal activity in aged KGN cells that had been continuously cultured for 3 months and beyond passage 15. (c) Expression of six genes between young and aged cells is detected using qPCR, with *GAPDH* as housekeeping gene. *t*-test was used to detect the significance. Data represent mean ± SD.; *n* = 3 in each group. ^∗^*p* < 0.05 and ^∗∗^*p* < 0.01.

Accumulation of senescent cells in tissues is a marker of chronological aging [27]. Since cells can be driven into senescence by repeated cell divisions, a senescent GC model was established by longtime culture with repeated passage of KGN cells. KGN cells that were freshly thawed and within passage 5 served as the young control, while aged cells refer to those that were continuously cultured for 3 months and beyond passage 15. The validity of this replication-induced cell aging model was confirmed by a significant increase in SA-*β*-gal activity, suggesting that the repeated cell divisions had induced cell senescence (Figures 6(a) and 6(b)). Then, the mRNA expressions of the six oxidative stress-related genes between young and aged cells were determined. Upregulated expression of all genes was observed in the aged cell group (*p* < 0.05) (Figure 6(c)).

### 3.6. Expressions of the Six Oxidative Stress-Related Genes of GCs in Young and Reproductively Aged Mice

**Figure 7:**
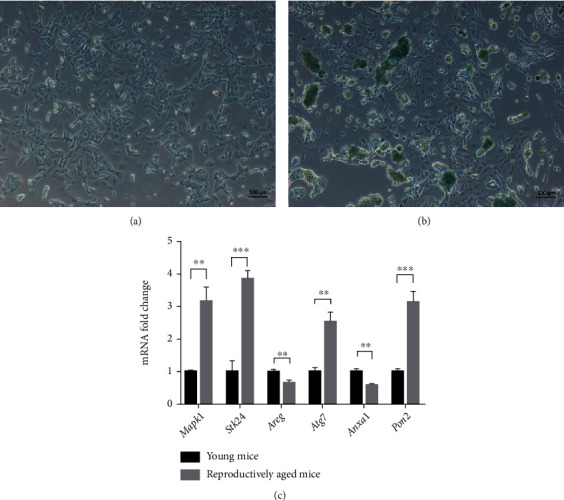
The expression of six oxidative stress-related genes between young and reproductively aged mice. (a) SA-*β*-gal activity of GCs from young mice. (b) SA-*β*-gal activity of GCs from reproductively aged mice. (c) Expression of the six genes between young and reproductively aged mouse GCs are detected using qPCR, with *Gapdh* as a housekeeping gene. *t*-test is used to detect the significance. Data represent mean ± SD.; *n* = 3 in each group. ^∗∗^*p* < 0.01 and ^∗∗∗^*p* < 0.001.

To further investigate the expressions of the six oxidative stress-related GC genes in ovarian aging, we examined mGCs. To determine whether GCs from young and reproductively aged mice exhibit significant cell senescence, SA-*β*-gal was assayed and showed significantly increased activity in reproductively aged mice (8-month old) compared to the young control (8-week old) (Figures 7(a) and 7(b)). Then, the mRNA expression of the six genes between young and reproductively aged mice was detected. Upregulated expression of *Mapk1*, *Stk24*, *Atg7*, and *Pon2* and downregulation of *Anxa1* and *Areg* were observed in the reproductively aged group (*p* < 0.01) (Figure 7(c)).

## 4. Discussion

In this study, we identified six oxidative stress-related genes (*MAPK1*, *STK24*, *AREG*, *ATG7*, *ANXA1*, and *PON2*) that are associated with ovarian aging and experimentally explored their expressions in the human GC cell line and mGCs (Table 2). Mitogen-activated protein kinases (MAPKs) are a family of serine/threonine protein kinases, which mediate fundamental biological processes and cellular responses to external stress signals, including oxidative stress [28]. In an ischemia/reperfusion mouse model, protection against myocardial injury can occur through inhibiting oxidative stress by targeting MAPK1 [29]. Similarly, oxidative stress can be ameliorated by inhibiting miR-125b-5p/*MAPK1* in humans, as well as mouse cell models of Parkinson's disease [30]. In our oxidative stress cell model, *MAPK1* is upregulated following menadione-induced oxidative stress in KGN cells, confirming its involvement in oxidative stress. However, the addition of antioxidant NAC did not reverse this upregulation, indicating *MAPK1* is an oxidative stress response regulator instead of a downstream effector, as implied by previous studies [28, 29]. On the other hand, MAPK1 has been found to be involved in the process, in which environmental enrichment prevented neuroplastic decline of the hippocampus during aging [31]. Consistently, our bioinformatic result shows that MAPK1 in GCs is differentially expressed between old and young NHPs and serves as a protective factor against the aging phenotype (Figure 4(b) and Table 1). Upregulation of *MAPK1* expressions is also seen in aged KGN cells and GCs in reproductively aged mice compared to their controls, thereby suggesting its potential critical role in ovarian aging.

*STK24*, which encodes serine/threonine kinase 24, plays a key role in mediating cellular demise in response to oxidative stress in a human colon carcinoma cell line [32]. Considering that STK24 functions upstream of MAPK, a signaling pathway can be activated by ROS to mediate GC senescence [33], as well as our findings showing that *STK24* is upregulated upon ROS induction and following prolonged passage of KGN cells, and also differentially expressed in GCs of old mice and NHPs, it is plausible that *STK24* has biological relevance for ovarian aging.

As the most abundant epidermal growth factor receptor (EGFR) ligand expressed in human GCs, *AREG* can be upregulated by luteinizing hormone (LH)/hCG [34]. A recent study has found that elevated expression of *AREG* in GCs contributes to the development of ovarian hyperstimulation syndrome (OHSS), a condition thought to involve oxidative stress [35, 36]. In addition, the findings of increased *AREG* expression in a senescent human prostate stromal cell line and epidermal melanocytes [37, 38] highlight the critical role of *AREG* in GC oxidative stress and senescence, which is also implied by our results.

It is generally accepted that autophagic activity declines with age, likely contributing to the accumulation of damaged macromolecules and organelles [39]. *ATG7*, a critical gene of the conventional autophagy pathway, is involved in the regulation of the antioxidant response and serves as a contributor to oxidative stress-induced senescence in human epidermal melanocytes [40]. In a genetically engineered melanoma mouse model, *Atg7* promotes tumor growth by limiting oxidative stress and senescence [41]. In line with these findings, all of our experiments, including bioinformatics and in vitro and in vivo studies, suggest that *ATG7* is closely associated with oxidative stress, cell senescence, and aging in GCs.

*ANXA1* is a calcium-dependent phospholipid binding protein encoding gene. In accordance with our result suggesting an increased expression upon oxidative stress induction, *ANXA1* was also upregulated in prostate cancer cell line under hypoxia, a condition that may lead to oxidative stress [42]. However, the link between *ANXA1* and aging has not been explored thus far. Considering the results showing significantly different expressions of *ANXA1* between young and aged KGN cells, mouse, and NHP GCs, the role of *ANXA1* in aging process deserves further investigation.

*PON2*, the oldest member of the paraoxonase family, encodes a mitochondrial protein that enhances mitochondrial function and exhibits antioxidative stress properties [43, 44]. The importance of the role of PON2 in a variety of aging processes and diseases associated with a high level of ROS has been recently highlighted, including cancer, cardiovascular diseases, neurodegeneration, and diabetes [43]. In addition, the fact that PON2 predominantly localizes in organelles with high oxidative stress also lends strong evidence to its critical role in preventing oxidative damage, likely scavenging ROS at the mitochondrial level or reducing generated ROS responding to endoplasmic reticulum stress [45, 46]. The positive correlation between *PON2* and aging and its role as a downstream effector upon oxidative stress is also confirmed in our bioinformatic analysis and oxidative stress cell model, respectively, indicating it as a potential crucial player during the process of ovarian aging.

Importantly, it should be noted that there are some inconsistences among the bioinformatic results and the in vitro and in vivo studies. For example, upregulation of *AREG* and *ANXA1* observed in old NHP GCs and senescent KGN cells is not observed in mGCs. The expression of *MAPK1* and *STK24* is downregulated in old NHP GCs but upregulated both in aged KGN cells and mGCs. These inconsistences can be attributed to the fact that the GCs used are slightly different in nature. First, there are important functional differences as well as distinct gene expression profiles between cumulus cells that are in direct contact with the oocyte and mural granulosa cells, which surround the follicle antrum [47, 48]. Second, GCs from follicles of different stages show differential gene expression profiles [47, 49]. While the NHP GCs analyzed contain both mural and cumulus GCs, as well as all stages of follicles, only mural mGCs from large antral follicles were isolated for gene expression detection in the in vivo study, and the KGN cells, which are derived from a stage-3 diagnosed GC tumor removed from a 63-year-old Japanese woman in 1984, are believed to have originated from a dominant follicle at the antral or preantral stage [50].

A recent study has identified two antioxidant genes (*IDH1* and *NDUFB10*) in GCs that were negatively correlated with age, as well as provided a comprehensive single-cell transcriptomic atlas of ovaries of young and old NHPs [19]. However, to the best of our knowledge, this is the first study aimed at establishing an oxidative stress-related signature, specifically in GCs, associated with ovarian aging. Since diseases are generally associated with expression change of a group of genes rather than an isolated gene, the construction of a gene signature based on a set of genes is more closed to the genetic nature of disease. In addition, by constructing a signature, key genes can be filtered out while in the meantime, maximal preservation of gene expression and clinical outcome information is achieved. Some innovations were also made in this study. Importantly, instead of studying bulk RNA-seq datasets, which mainly reflect the averaged gene expression across thousands of cells, we make use of the single-cell RNA resource and platform provided by Wang et al. [19], which allows exploration of gene expression profiles at the single-cell level and reveals cell-to-cell gene expression variability. In addition, we independently validated the NHP oxidative stress-related gene signature in a human GC mRNA dataset (GSE81579), with good performance in differentiating old and young women, thereby further supporting the discriminatory capacity of the signature. Finally, *in vitro* and *in vivo* studies were carried out to characterize the expression levels of these six genes upon oxidative stress, senescence, and aging, thus providing experimental evidence for the critical roles of the six genes of GCs in ovarian aging.

Nevertheless, several limitations in this study should be acknowledged. First, the six oxidative stress-related genes were only experimentally investigated in a human granulosa cell line and mGCs, although prior bioinformatic analysis based on public scRNA-seq was conducted to direct the exploration of the genetic nature of GCs contributing to ovarian aging. Hence, if clinical application is expected, the discriminatory capacity of our oxidative stress-related signature should be prospectively verified in a large clinical cohort with human GCs collected. Second, although this study has confirmed the aberrant expression of the six oxidative stress-related genes in both KGN cells and mGCs, the in-depth molecular mechanisms of how each of these oxidative stress-related genes affect GC senescence as well as ovarian aging need to be explored further. Finally, to be more precise, GCs should be subdivided in future studies according to their different locations and functions (mural and cumulus) and stages (primordial, primary, secondary, and tertiary), since their functions and gene expression profiles are different.

In conclusion, this study provides a measurable assessment of female ovarian aging by establishing a six oxidative stress-related gene signature based on a single-cell transcriptomic primate database, followed by validation in a human bulk-RNA seq database. Importantly, current efforts are being invested for the expression of the six critical oxidative stress-related genes in models of oxidative stress, replication-induced senescence, and reproductively aged mice. The identification of these potential oxidative stress-related biomarkers adds new insights into the molecular contribution of GC oxidative stress to ovarian aging.

## Figures and Tables

**Table 1:** Fold change of gene expression of young NHPs compared to the old and the respective factor to the phenotype of being old or young.

Gene	logFC	*p* value	FDR	Factor
*MAPK1*	2.1886068	3.43*E* − 07	2.99*E* − 05	1.81*E* − 04
*STK24*	1.79691868	7.02*E* − 05	0.00175984	6.43*E* − 04
*ATG7*	2.02357625	0.00015649	0.00334927	1.84*E* − 03
*ANXA1*	-1.0269562	5.29*E* − 05	0.00137536	−1.30*E* − 03
*PON2*	-1.4895272	0.00292571	0.02825404	−3.19*E* − 04
*AREG*	-2.402029	0.00086888	0.01188395	−6.77*E* − 05

logFC: log2(fold change); FDR: false discovery rate.

**Table 2:** Expressions of the six oxidative stress-related genes in old NHPs, oxidative stress-induced KGN cells, senescent KGN cells, and reproductively aged mice compared to their controls, respectively.

	Old NHPs (public data)	Oxidative stress-induced KGN cells	Senescent KGN cells	Reproductively aged mice
*MAPK1/Mapk1*	↓	↑	↑	↑
*STK24/Stk24*	↓	↑	↑	↑
*AREG/Areg*	↑	↓	↑	↓
*ATG7/Atg7*	↓	↑	↑	↑
*ANXA1/Anxa1*	↑	↑	↑	↓
*PON2/Pon2*	↑	↑	↑	↑

## Data Availability

The data used to support the finding of this study are available from the corresponding author upon request.

## Supplementary Materials

Supplementary 1 Table S1: the primers used for qRT-PCR.

Supplementary 2 Table S2: GO and KEGG pathways associated with the signature.
