# Supplementary material for: An Oxidative Stress-Related Gene Signature in Granulosa Cells Is Associated with Ovarian Aging
Source: Oxid Med Cell Longev. 2022 Nov 3;2022:1070968. doi: 10.1155/2022/1070968 (PMC9713466; doi:10.1155/2022/1070968)
Supplement: Supplementary 1 — Table S1: the primers used for qRT-PCR. [file 1070968.f1.docx]

Table S1 The primers used for qRT-PCR

| GENE | PRIMER SEQUENCES | |
| --- | --- | --- |
|  | Forward sense（5'-3'） | Reverse antisense（5'-3'） |
| *AREG* | TGAGATGTCTTCAGGGAGTG | AGCCAGGTATTTGTGGTTCG |
| *ATG7* | CGTTGCCCACAGCATCATCTTC | CACTGAGGTTCACCATCCTTGG |
| *ANXA1* | GCGAAACAATGCACAGCGTCAAC | CAACCTCCTCAAGGTGACCTGT |
| *PON2* | CCAGAAGCTCTTCGTGTATGACC | GAACTTCCTTGGAGAACAGACCC |
| *MAPK1* | ACACCAACCTCTCGTACATCGG | TGGCAGTAGGTCTGGTGCTCAA |
| *STK24* | GCCTGTTTGAATAAGGAGCCGAG | CCTTCCATCTCTTGTACCTGTCG |
| *GAPDH* | ACAACTTTGGTATCGTGGAAGG | GCCATCACGCCACAGTTTC |
| *Atg7* | CCTGTGAGCTTGGATCAAAGGC | GAGCAAGGAGACCAGAACAGTG |
| *Anxa1* | TGTATCCTCGGATGTTGCTGCC | CCATTCTCCTGTAAGTACGCGG |
| *Pon2* | ACAGAGGCTCTTCGTGTACCAC | CAGAACTTCCCTGGAGGACAGA |
| *Areg* | GCAGATACATCGAGAACCTGGAG | CCTTGTCATCCTCGCTGTGAGT |
| *Mapk1* | TCAAGCCTTCCAACCTCCTGCT | AGCTCTGTACCAACGTGTGGCT |
| *Stk24* | GGTCATCAAGCAGTCAGCCTAC | TGCAGCTCAGAATGTGGTGGCT |
| *Gapdh* | CATCACTGCCACCCAGAAGACTG | ATGCCAGTGAGCTTCCCGTTCAG |
