# Supplementary material for: An Oxidative Stress-Related Gene Signature in Granulosa Cells Is Associated with Ovarian Aging
Source: Oxid Med Cell Longev. 2022 Nov 3;2022:1070968. doi: 10.1155/2022/1070968 (PMC9713466; doi:10.1155/2022/1070968)
Supplement: Supplementary 2 — Table S2: GO and KEGG pathways associated with the signature. [file 1070968.f2.docx]

Table S2 GO and KEGG pathways associated with the signature

| NAME | SIZE | ES | NES | NOM p-val | FDR q-val |
| --- | --- | --- | --- | --- | --- |
| GO_NADH_DEHYDROGENASE_ACTIVITY | 44 | -0.65663 | -1.73468 | 0.030769 | 0.205806 |
| GO_RESPIRATORY_CHAIN_COMPLEX | 70 | -0.66269 | -1.70764 | 0.032946 | 0.20825 |
| GO_CELLULAR_METABOLIC_COMPOUND_SALVAGE | 23 | -0.50608 | -1.66594 | 0.017647 | 0.212343 |
| GO_CIS_TRANS_ISOMERASE_ACTIVITY | 33 | -0.59553 | -1.70283 | 0.022901 | 0.212379 |
| GO_U12_TYPE_SPLICEOSOMAL_COMPLEX | 24 | -0.63699 | -1.648 | 0.044807 | 0.212396 |
| GO_MITOCHONDRIAL_TRANSMEMBRANE_TRANSPORT | 64 | -0.48255 | -1.73543 | 0.007619 | 0.212516 |
| GO_PROTEIN_IMPORT_INTO_MITOCHONDRIAL_MATRIX | 15 | -0.62328 | -1.70831 | 0.029014 | 0.212806 |
| GO_RIBONUCLEOPROTEIN_COMPLEX_BIOGENESIS | 376 | -0.46179 | -1.63858 | 0.062241 | 0.21287 |
| GO_NCRNA_PROCESSING | 326 | -0.3989 | -1.63997 | 0.073705 | 0.213456 |
| GO_ANAPHASE_PROMOTING_COMPLEX | 17 | -0.50554 | -1.64934 | 0.03681 | 0.213634 |
| GO_VIRAL_GENE_EXPRESSION | 182 | -0.71994 | -1.69684 | 0.052941 | 0.213638 |
| GO_PYRIMIDINE_NUCLEOSIDE_METABOLIC_PROCESS | 19 | -0.5853 | -1.68868 | 0.015414 | 0.214127 |
| GO_NUCLEAR_TRANSCRIBED_MRNA_CATABOLIC_PROCESS | 189 | -0.69606 | -1.65082 | 0.0625 | 0.214186 |
| GO_OXIDOREDUCTASE_ACTIVITY_ACTING_ON_A_SULFUR_GROUP_OF_DONORS | 45 | -0.47388 | -1.6584 | 0.017045 | 0.214869 |
| GO_OXIDATIVE_PHOSPHORYLATION | 110 | -0.56319 | -1.69891 | 0.031365 | 0.215104 |
| GO_TRANSLATIONAL_INITIATION | 175 | -0.7674 | -1.66013 | 0.039216 | 0.215347 |
| GO_GLYCOSYL_COMPOUND_CATABOLIC_PROCESS | 25 | -0.56121 | -1.66644 | 0.013672 | 0.215494 |
| GO_ESTABLISHMENT_OF_PROTEIN_LOCALIZATION_TO_ORGANELLE | 451 | -0.491 | -1.66219 | 0.054217 | 0.215659 |
| GO_NUCLEOSIDE_CATABOLIC_PROCESS | 17 | -0.60425 | -1.64049 | 0.019417 | 0.215746 |
| GO_BINDING_OF_SPERM_TO_ZONA_PELLUCIDA | 16 | -0.62998 | -1.65582 | 0.012526 | 0.216307 |
| GO_RIBOSOME | 207 | -0.69249 | -1.65136 | 0.045817 | 0.216883 |
| GO_SPLICEOSOMAL_SNRNP_ASSEMBLY | 36 | -0.52899 | -1.73725 | 0.017893 | 0.217155 |
| GO_RRNA_METABOLIC_PROCESS | 196 | -0.48996 | -1.70932 | 0.056795 | 0.217317 |
| GO_U2_SNRNP | 18 | -0.69856 | -1.65294 | 0.045908 | 0.217716 |
| GO_POLYSOME | 66 | -0.72213 | -1.64306 | 0.03937 | 0.217901 |
| GO_SPLICEOSOMAL_TRI_SNRNP_COMPLEX | 29 | -0.55192 | -1.66725 | 0.070565 | 0.218259 |
| GO_U1_SNRNP | 17 | -0.72873 | -1.64093 | 0.014523 | 0.218482 |
| GO_THIOLESTER_HYDROLASE_ACTIVITY | 27 | -0.47702 | -1.68908 | 0.001898 | 0.218768 |
| GO_AEROBIC_RESPIRATION | 73 | -0.54679 | -1.63383 | 0.062622 | 0.218953 |
| GO_NUCLEOBASE_CONTAINING_SMALL_MOLECULE_CATABOLIC_PROCESS | 30 | -0.50341 | -1.72455 | 0.013672 | 0.2193 |
| GO_ORGANELLAR_RIBOSOME | 83 | -0.51001 | -1.66757 | 0.069444 | 0.222338 |
| GO_PRECATALYTIC_SPLICEOSOME | 49 | -0.51866 | -1.68977 | 0.06135 | 0.222851 |
| GO_PROTEIN_TARGETING_TO_MITOCHONDRION | 83 | -0.44859 | -1.70959 | 0.017241 | 0.223521 |
| GO_MITOCHONDRIAL_ELECTRON_TRANSPORT_NADH_TO_UBIQUINONE | 52 | -0.63131 | -1.73795 | 0.034549 | 0.224885 |
| GO_ORGANIC_CYCLIC_COMPOUND_CATABOLIC_PROCESS | 470 | -0.51077 | -1.71543 | 0.047619 | 0.225457 |
| GO_MITOCHONDRIAL_TRANSLATION | 127 | -0.4605 | -1.62553 | 0.080925 | 0.225841 |
| GO_PEPTIDASE_COMPLEX | 81 | -0.49809 | -1.67789 | 0.056974 | 0.225955 |
| GO_NEGATIVE_REGULATION_OF_UBIQUITIN_DEPENDENT_PROTEIN_CATABOLIC_PROCESS | 43 | -0.43674 | -1.62353 | 0.054159 | 0.226354 |
| GO_PROTEIN_TARGETING_TO_MEMBRANE | 159 | -0.75619 | -1.66787 | 0.02549 | 0.226485 |
| GO_SPERM_EGG_RECOGNITION | 18 | -0.61037 | -1.62804 | 0.010183 | 0.227253 |
| GO_RIBOSOME_BIOGENESIS | 260 | -0.51037 | -1.67944 | 0.047325 | 0.228085 |
| GO_CHAPERONE_MEDIATED_PROTEIN_FOLDING | 48 | -0.4663 | -1.66968 | 0.013752 | 0.2282 |
| GO_POSITIVE_REGULATION_OF_MONOOXYGENASE_ACTIVITY | 16 | -0.63843 | -1.62574 | 0.00404 | 0.228862 |
| GO_NUCLEAR_UBIQUITIN_LIGASE_COMPLEX | 37 | -0.47881 | -1.71722 | 0.008048 | 0.229188 |
| GO_ELECTRON_TRANSPORT_CHAIN | 144 | -0.50958 | -1.67145 | 0.031895 | 0.229717 |
| GO_OXIDOREDUCTASE_ACTIVITY_ACTING_ON_NAD_P_H_QUINONE_OR_SIMILAR_COMPOUND_AS_ACCEPTOR | 54 | -0.63491 | -1.71003 | 0.032381 | 0.229832 |
| GO_RIBOSOMAL_SMALL_SUBUNIT_BIOGENESIS | 62 | -0.62018 | -1.67371 | 0.053571 | 0.230169 |
| GO_RESPIRATORY_CHAIN_COMPLEX_IV_ASSEMBLY | 23 | -0.64092 | -1.73853 | 0.006173 | 0.233184 |
| GO_RNA_CATABOLIC_PROCESS | 341 | -0.59828 | -1.74621 | 0.041833 | 0.236516 |
| GO_TRANSLATIONAL_TERMINATION | 101 | -0.45319 | -1.61471 | 0.094118 | 0.238034 |
| GO_PROTEIN_TARGETING | 352 | -0.56676 | -1.74 | 0.037328 | 0.240002 |
| GO_CYTOPLASMIC_TRANSLATION | 90 | -0.69095 | -1.61508 | 0.052525 | 0.240659 |
| GO_NADH_DEHYDROGENASE_COMPLEX | 46 | -0.65611 | -1.74697 | 0.026923 | 0.246314 |
| GO_ANTIGEN_PROCESSING_AND_PRESENTATION_OF_PEPTIDE_ANTIGEN_VIA_MHC_CLASS_I | 78 | -0.50928 | -1.60584 | 0.070588 | 0.246375 |
| GO_RIBOSOMAL_SUBUNIT | 172 | -0.73705 | -1.59922 | 0.047809 | 0.248111 |
| GO_NEGATIVE_REGULATION_OF_RNA_SPLICING | 24 | -0.60855 | -1.60026 | 0.076923 | 0.24889 |
| GO_DISULFIDE_OXIDOREDUCTASE_ACTIVITY | 30 | -0.53037 | -1.60608 | 0.04 | 0.249246 |
| GO_EXON_EXON_JUNCTION_COMPLEX | 19 | -0.50815 | -1.60293 | 0.049213 | 0.249252 |
| KEGG_DRUG_METABOLISM_OTHER_ENZYMES | 15 | -0.64604 | -1.85103 | 0.005859 | 0.090041 |
| KEGG_PARKINSONS_DISEASE | 95 | -0.6131 | -1.64033 | 0.070313 | 0.192957 |
| KEGG_FATTY_ACID_METABOLISM | 30 | -0.6329 | -1.66911 | 0.011952 | 0.193281 |
| KEGG_SNARE_INTERACTIONS_IN_VESICULAR_TRANSPORT | 33 | -0.41958 | -1.68799 | 0.03854 | 0.219245 |
| KEGG_ANTIGEN_PROCESSING_AND_PRESENTATION | 36 | -0.63868 | -1.59388 | 0.043893 | 0.238005 |
| KEGG_CARDIAC_MUSCLE_CONTRACTION | 40 | -0.53666 | -1.51521 | 0.045098 | 0.242487 |
| KEGG_ALZHEIMERS_DISEASE | 122 | -0.43782 | -1.54135 | 0.077381 | 0.244528 |
| KEGG_PROTEASOME | 40 | -0.64683 | -1.52448 | 0.101365 | 0.249813 |
